# Chemical Composition and Antifungal Activity of *Ocimum basilicum* L. Essential Oil

**DOI:** 10.3889/oamjms.2015.082

**Published:** 2015-07-10

**Authors:** Neveen Helmy Abou El-Soud, Mohamed Deabes, Lamia Abou El-Kassem, Mona Khalil

**Affiliations:** 1 *Complementary Medicine Department, Medical Researches Division, National Research Center, El-Behouth Street, Dokki, 12311 Cairo, Egypt*; 2 *Food Toxicology and Contaminants Department, National Research Center, El-Behouth Street, Dokki, 12311 Cairo, Egypt*; 3 *Pharmacognosy Department - National Research Center- El-Behouth Street, Dokki, 12311 Cairo, Egypt*; 4 *Biochemistry Department – Gizan University - K.S.A., Cairo, Egypt*

**Keywords:** *Ocimum basilicum* L. essential oil, chemical composition, antifungal activity, aflatoxins B1, *Aspergillus flavus*

## Abstract

**BACKGROUND:**

The leaves of *Ocimum basilicum* L. (basil) are used in traditional cuisine as spices; its essential oil has found a wide application in perfumery, dental products as well as antifungal agents.

**AIM:**

To assess the chemical composition as well as the *in vitro* antifungal activity of *O. basilicum* L. essential oil against *Aspergillus flavus* fungal growth and aflatoxin B1 production.

**MATERIAL AND METHODS:**

The essential oil of *O. basilicum* was obtained by hydrodistillation and analysed using gas chromatography (GC) and GC coupled with mass spectrometry (GC/MS). The essential oil was tested for its effects on *Aspergillus flavus* (*A. flavus*) mycelial growth and aflatoxin B1 production in Yeast Extract Sucrose (YES) growth media. Aflatoxin B1 production was determined by high performance liquid chromatography (HPLC).

**RESULTS:**

Nineteen compounds, representing 96.7% of the total oil were identified. The main components were as follows: linalool (48.4%), 1,8-cineol (12.2%), eugenol (6.6%), methyl cinnamate (6.2%), α-cubebene (5.7%), caryophyllene (2.5%), β-ocimene (2.1%) and α-farnesene (2.0%). The tested oil showed significant antifungal activity that was dependent on the used oil concentration. The complete inhibition of *A. flavus* growth was observed at 1000 ppm oil concentration, while marked inhibition of aflatoxin B1 production was observed at all oil concentrations tested (500, 750 and 1000 ppm).

**CONCLUSION:**

These results confirm the antifungal activities of *O. basilicum* L. oil and its potential use to cure mycotic infections and act as pharmaceutical preservative against *A. flavus* growth and aflatoxin B1 production.

## Introduction

Basil has been traditionally used as a food spice, in perfumery and medical industries [1]. Ancient Egyptians used it in their medical prescriptions [2]. In folk medicine, the leaves and flowering tops of the plant are prescribed as carminative, galactogogue, stomachic, antispasmodic [3]. However, recently the potential uses of *O. basilicum* essential oil, particularly as antimicrobial [4], antifungal [5], antioxidant [6] and larvicidal [7] have also been investigated.

The genus *Ocimum*, Lamiaceae, collectively called basil, has long been acclaimed for its diversity. *Ocimum* comprises more than 30 species of herbs and shrubs from the tropical and subtropical regions of Asia, Africa, Central and South America, but the main center of diversity appears to be Africa [8].

The *O. basilicum* essential oils exhibit a wide and varying array of chemical compounds, depending on variations in chemotypes, leaf and flower colours, aroma and origin of the plants [9]. The chief constituents include chavicol methyl ether or estragole, linalool and eugenol [10].

Recently due to the alarming increase in the rate of infection by antibiotic resistant microorganisms, immense clinical problems have been encountered in treatment of infectious diseases. Extensive research work has to be done to eliminate this serious problem. Fortunately, natural products particularly essential oils can participate effectively in this scope. One of these microorganisms is *A. favus* fungus which produces secondary metabolites known as aflatoxins that are potentially harmful to crops, animals and humans. The aflatoxins B1, B2, G1 and G2 are the major four toxins amongst at least 16 structurally related toxins. Aflatoxin B1 is particularly important since it is the most toxic and potent hepatocarcinogenic natural compound ever characterized [11].

*A. flavus* causes a broad spectrum of diseases in humans, ranging from hypersensitivity reactions to invasive infections associated with angioinvasion. *A. flavus* is the second leading cause of invasive and noninvasive aspergillosis [12]. Multiple resistances to known antifungal drugs with high mortality rate have been reported. This increases the need to explore natural drugs of high potency and effectiveness against *A. flavus* and their aflatoxins.

In this study, the chemical composition and effects of essential oil of *Ocimum basilicum* L. (family *Apiaceae or Umbelliferae)* on growth and aflatoxin B1 production of *A. flavus* were evaluated.

## Material and Methods

The protocol of the study was reviewed and approved by Ethical Committee of National Research Center.

Authors declare that there is no actual or potential conflict of interest for this work.

### Plant Material

Leaves of *Ocimum basilicum* L. were purchased from local markets and authenticated in the herbarium of Faculty of Science, Cairo University and National Research Center, Egypt.

**Extraction of Essential Oil:** 1 kg of *Ocimum basilicum* L. leaves were subjected to hydrodistillation. The volatile oil then collected and dried in desiccators over anhydrous CaSO_4_. The volatile oil sample was kept in dark bottle for GC and GC/MS (mass spectrometry) analyses.

**Analysis of the Essential Oil GC:** The essential oil was analysed on Perkin-Elmer gas chromatograph model 8700, equipped with HP-5MS capillary column (30 m × 0.25 mm, film thickness 0.25 μm)programming from 80ºC (3 min) to 220ºC (10 min). Helium as a carrier gas (1.5 ml/min). Injection in split mode (1:100).

**GC/MS:** The essential oil was also analysed on Agilent-Technologies 6890N network gas chromatographic (GC) system (Little Falls, California, USA), equipped with HP-5 MS capillary column (30 m × 0.25 mm, film thickness 0.25 μm) programming from 80ºC (3 min) to 220ºC (10 min). Helium as a carrier gas (1.5 ml/min). Injection in split mode (1:100) [13, 14].

**Identification of Compounds:** Compounds were identified by computer search using their mass spectra either with the known components or published spectra and by comparison of their retention indices with those of known compounds [15, 16].

### Fungal inoculum preparation

*A. flavus* strain (ATCC 16872), was used as an aflatoxigenic strain for in vitro experiments. They were kept on potato-dextrose-agar (PDA) media and were incubated at 25ºC for 10 days. From single spore cultures of *A. flavus* grown on PDA media, the spores were harvested from the surface of the agar plates by washing off the surface of the slant with 10 ml of sterile 0.1% Tween 80 (Merck, Germany) solution to obtain a concentration of 106 spores/ml, and were utilized on the same day.

### Antifungal activity of essential oils

Fifteen ml of YES medium in 4 flasks (250 ml) was autoclaved at 120ºC for 15 minutes. 1 ml of a suspension of spores (105 spores) of the toxigenic *A. flavus* strain was inoculated in each flask with either 50 µl (500 ppm), 100 µl (750 ppm), or 150 µl (1000 ppm), of basil essential oil or without essential oil (control). The flasks were incubated in the dark for 14 days at 25 ºC. After the incubation period, the growth of the aflatoxigenic fungi *A. flavus* in all flasks was visually examined.

### Determination of mycelial weight

Extraction of aflatoxins produced in the YES culture was carried out [17] by harvesting the mycelium of each flask through filtration in Whatman paper (No. 4) then extraction by 100 ml chloroform. The chloroform extract was then dried by adding anhydrous sodium sulfate and evaporated to dryness under nitrogen at temperature below 60ºC. The net dry weight of mycelia was determined. The dry film was then used for the detection of aflatoxins.

### Derivatization

Two hundred µl hexane were added to the clean up dry film of control and tested samples followed by 50 µl trifluroacetic acid (TFA) and mixed by vortex vigorously for 30 seconds. The mixture was left to stand for 5 minutes. Then, 450 ml water – acetonitrile (9 +1 v/v) were added and mixed well for 30 seconds. The mixture was left to stand for 10 minutes to form two separate layers. The lower aqueous layer was used for HPLC analysis [18].

HPLC analysis: Using HPLC [Model 1525, Waters 1500 Rheodyne manual injector, 2475 Multi-Wavelength Fluorescence Detector, and a data workstation with software Breeze 2. A phenomenex C18 (250x 4.6 mm i.d), 5 μm from Waters corporation (USA) was used], the mixed solutions of control as well as tested extracts were filtered through a 0.22 mm membrane filter and loaded (20 ml) into a 20 ml injection loop.

**Figure 1:**
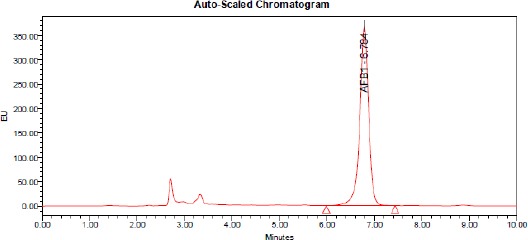
*HPLC of aflatoxin B1 produced by A. flavus (control)*.

**Figure 2:**
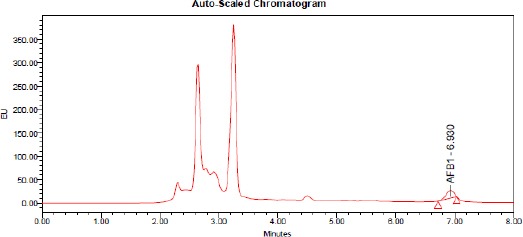
*HPLC of aflatoxin B1 produced by A. flavus treated by O. basilicum L. essential oil at 500 ppm concentration*.

**Figure 3:**
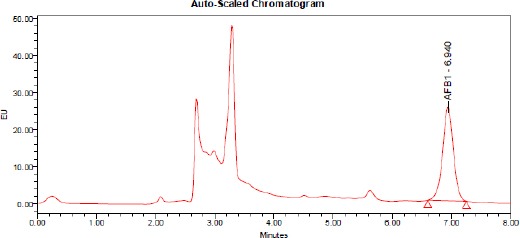
*HPLC of aflatoxin B1 produced by A. flavus treated by O. basilicum L. essential oil at 750 ppm concentration*.

**Figure 4:**
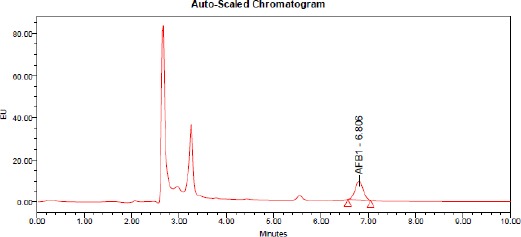
*HPLC of aflatoxin B1 produced by A. flavus treated by O. basilicum L. essential oil at 1000 ppm concentration*.

The elution order of the four aflatoxins was G2, B2, G2a (G1 derivative), B2a (B1 derivative). AFs contents in samples were calculated from chromatographic peak areas using the standard curve (Fig. 1, 2, 3, 4).

The experiments were replicated three times. The inhibitory % of fungal growth and aflatoxin production was calculated according to the equation:

Inhibitory % = (control- treatment /control ×100).

### Statistical analysis

All data from three independent replicate trials were subjected to statistical analysis using statistical software (SPSS, 10.0; Chicago, USA). Data are reported as means ± standard deviations. The significant differences between mean values were determined by independent sample t-test (*P* < 0.05).

## Results

Qualitative and quantitative chemical compositional data of *Ocimum basilicum* oil is given in Table 1.

**Table 1:** *Ocimum basilicum* L. essential oil components

Component		Composition %
1	α-Pinene	0.7
2	Camphene	1
3	β-Pinene	1.5
4	β-Myrcene	1.2
5	1, 8- Cineol	12.2
6	β-Ocimene	2.1
7	γ-Terpin	1.3
8	Linalool	48.4
9	Camphor	0.8
10	Myrtenol	1.5
11	α-Cubebene	5.7
12	Eugenol	6.6
13	Methyl cinnamate	6.3
14	Caryophyllene	2.5
15	Azulene	1.6
16	α-Farnesene	2
17	Germacrene B	1.9
18	Germacrene D	0.9
19	Naphthalene	0.5
Total %		96.7

Regarding the antifungal activity of the tested oil, results show that both fungal growth and aflatoxin biosynthesis were significantly suppressed by *O. basilicum* essential oil compared to control (Table 2).

**Table 2:** Effect of different concentrations of *Ocimum basilicum L.* essential oil on the mycelia dry weight and aflatoxin B1 production

Treatment	Concentration (*ppm*)	Mycelia dry weight (*g/ml*)a	Aflatoxin B1 (*lg/ml*)a
*Ocimum basilicum L.*	500	15.35 ± 1.53 ^a,c^	0.68 ± .053 ^a,c^
	750	5.333 ± 1.154 ^b^	0.516 ± .020
	1000	.000 ± .000	0.496 ± .015
Positive control		71.666 ± 1.527	25.808 ± .599

a significant differences between concentration 500 & 750

b significant differences between concentration 750 & 1000

c significant differences between concentration 500 & 1000 in the same column. a Data are means of triplicates (± standard deviation).

**Figure 5:**
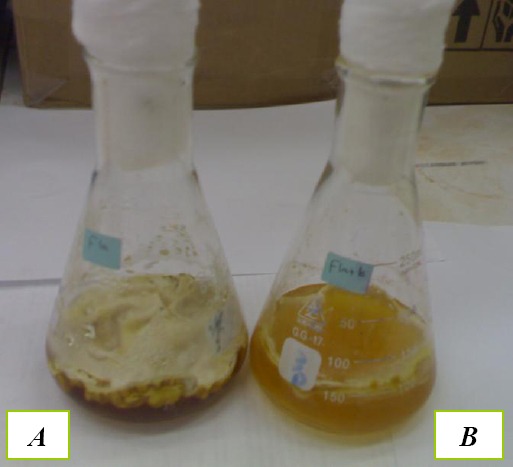
*Flask A, control flask showing growth of A. flavus. Flask B, containing O.basilicum L. essential oil at concentration of 1000 ppm showing no growth of A. flavus*.

There was no mycelia growth of *A. flavus* fungus at 1000 ppt concentration of the tested oil (Fig 5).

**Figure 6:**
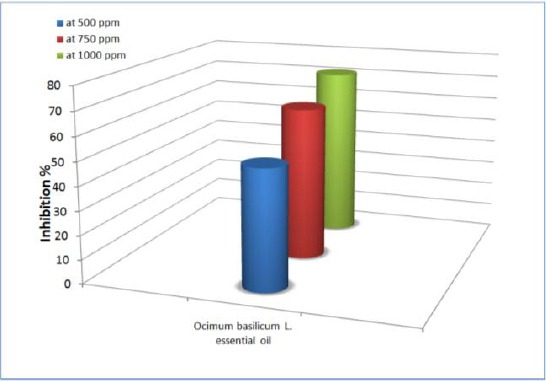
*The inhibitory % of O. basilicum L essential oil on mycelia growth of A. flavus*.

**Figure 7:**
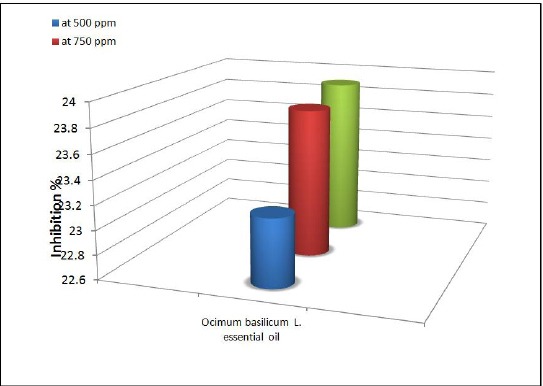
*The inhibitory % of O. basilicum L essential oil on aflatoxin B1 of A. flavus*.

The inhibitory % is one of the elements necessary for the evaluation of the effectiveness of an extract. The inhibitory % of the oil increased in proportion to its concentration (Fig. 6, 7).

## Discussion

This study confirms the antifungal properties of *O. basilicum* L. essential oil against *A. flavus* mycelial growth and aflatoxin B1 production. The tested oil showed significant antifungal activity that was dependent on the used oil concentration. The complete inhibition of *A. flavus* growth was observed at 1000 ppm oil concentration, while marked inhibition of aflatoxin B1 production was observed at all oil concentrations tested (500, 750 and 1000 ppm).

In agreement with our findings, a number of studies report on strong antifungal action of basil essential oil. Doube et al. [20] using the agar plate method, showed that basil oil, in a concentration of 1.5 ml/l inhibited completely the growth of 22 species of molds, including the aflatoxigenic strains *Aspergillus parasiticus* and *A. flavus*.

Another study on *Candida albicans* and *A. flavus* [21] reported that basil oil inhibited completely the growth of these organisms at a concentration of 5000 ppm, during 7 days of incubation, using microdilution method. Soliman and Badeaa [22] found that basil oil acts as a fungistatic agent against *F. verticillioides* in a concentration of 2000 ppm, and as a fungicid agent in concentration of 3000 ppm. While complete growth inhibition of *F. verticillioides* was noticed in another study at concentrations higher than 2.7 μL/mL [23].

Many researchers reported that essential oil of plants was able to inhibit both growth and/or mycotoxin production [24, 25]. The antiaflatoxigenic actions of essential oil may be related to inhibition of the ternary steps of aflatoxin biosynthesis involving lipid peroxidation and oxygenation [26]. In addition essential oils protect the cells from harmful impact of aflatoxins through two possible mechanisms firstly by reducing DNA binding formation of aflatoxins, secondly by reacting with ROS increased by aflatoxins [27].

Some researchers reported that there is a relationship between the chemical structures of the most abundant compounds in the essential oils and the antimicrobial or antifungal activities [28]. Compositional analysis of *O. basilicum* essential oil in our study showed that compounds such as linalool, 1,8-cineol, eugenol, methyl cinnamate and α-cubebene were among the main components present. The antimicrobial activity of an essential oil is attributed mainly to its major compounds. However, the synergistic or antagonistic effect of one compound in minor percentage in the mixture has to be considered [29].

Early researches addressed linalol, estragol, eugenol and methyl cinnamate as the major antimicrobial and antifungal components of basil extracts and essential oil [30]. Some pointed to strong antifungal effect of oil which contained estragol as the main component on the growth of *Aspergillus niger*, *A. ochraceus*, and *Fusarium culmorum* (inhibition growth of 71.0 to 94.76%) [31], others found linalol and estragol to be more efficient against *R. nigricans* (100% of inhibition), compared to eugenol (38.1% of inhibition) [32]. Eugenol was found to exhibit stronger inhibition towards *F. oxysporum* (100% of inhibition), in contrast to linalool and estragol, where the inhibition values were 26.4 and 30.3%, respectively [4].

Our findings suggest that *O. basilicum* L. at concentration of 1000 ppt could supress *A. flavus* fungal growth and inhibit markedly aflatoxin B1 production which could be attributed to its rich content of linalool, 1,8-cineol and eugenol.

In conclusion, these results confirm the antifungal activities of *O. basilicum* L. oil and its potential use to cure mycotic infections and act as pharmaceutical preservative against *A. flavus* growth and aflatoxin B1 production.
